# Sociodemographic Correlates and Mental Health Comorbidities in Adolescents With Social Anxiety: The Young-HUNT3 Study, Norway

**DOI:** 10.3389/fpsyg.2021.663161

**Published:** 2021-04-16

**Authors:** Ingunn Jystad, Ottar Bjerkeset, Tommy Haugan, Erik R. Sund, Jonas Vaag

**Affiliations:** ^1^Faculty of Nursing and Health Science, Nord University, Levanger, Norway; ^2^Department of Public Health and Nursing, Faculty of Medicine and Health Science, Norwegian University of Science and Technology, Trondheim, Norway; ^3^Department of Mental Health, Norwegian University of Science and Technology, Trondheim, Norway; ^4^Department of Public Health and Nursing, HUNT Research Centre, Norwegian University of Science and Technology, Trondheim, Norway; ^5^Levanger Hospital, Nord-Trøndelag Hospital Trust, Levanger, Norway; ^6^Department of Psychology, Faculty of Social and Educational Sciences, Norwegian University of Science and Technology, Trondheim, Norway

**Keywords:** social anxiety disorder, adolescence, ADIS-C, self-report, sociodemograhics, comorbidity, HUNT–study

## Abstract

Social anxiety is highly prevalent in adolescents and is often associated with great individual suffering and functional impairment. Psychiatric comorbidity is common and further adds to this burden. The purposes of this study were: (1) to describe the occurrence of diagnosed and self-reported social anxiety among 8,199 Norwegian adolescents aged 13–19 years who participated in the population-based Young-HUNT3 study (2006–2008); (2) to examine associations between sociodemographic characteristics and different subgroups of social anxiety; and (3) to describe the psychiatric health comorbidities among adolescents diagnosed with social anxiety disorder (SAD). In total, 388 (5.9%) of the adolescents screened positive for SAD and were invited into a diagnostic interview, performed by professional nurses, using Anxiety Disorders Interview Schedule for DSM IV: child version (ADIS-C) (response rate = 54.6%). A SAD diagnosis was indicated in 106 individuals (50% of the interview subjects), and more than two-thirds of the adolescents diagnosed with SAD had one or more comorbid psychiatric disorders. Higher mean scores of self-reported social anxiety symptoms, poor self-rated health, sleep problems, poor family economic situation, low physical activity, and having sought professional help within the last year were associated with higher odds of being in the screening positive subgroup. Screening positive subjects who did not meet for a diagnostic interview did not differ notably from the rest of the screening positive group in terms of these sociodemographic characteristics. Based on our results and the fact that individuals with social anxiety often fear interview situations, the use of ADIS-C, screening questions and self-reports seem to be sufficient when aiming to identify epidemiologically representative cohorts of adolescents at risk of social anxiety.

## Introduction

Social anxiety disorder (SAD) is defined by a “marked and persistent fear of one or more social or performance situations in which the person is exposed to unfamiliar people or to possible scrutiny by others” (American Psychiatric Association, 2000, p 456). Since these situations involve a significant amount of emotional distress, those affected tend to avoid them. With a global lifetime risk of 4% (Stein et al., 2017), SAD is one of the most common anxiety disorders (Stein and Stein, 2008). It appears to be more prevalent among women than men (Asher et al., 2017), as well as more often in Western than Eastern countries, although this is questioned due to cultural differences in symptom presentation (Hofmann et al., 2010; Wong et al., 2019). The condition tends to have an early debut (Fehm et al., 2005): Median debut age is 13 years, and 90% of cases develop before the age of 23 (Kessler et al., 2005). When untreated, the condition typically persists (Stein et al., 2017), and often runs a chronic (Fehm et al., 2005) and remitting course, swinging above and below the diagnostic threshold (Beesdo-Baum et al., 2012). Psychiatric comorbidities are common and further adds to the burden. Previous studies of adolescents with SAD have reported a comorbidity rate for additional psychiatric health problems of around 60–70%, including both single and multiple conditions (Wittchen et al., 1999; Ranta et al., 2009). The proportion of adults with both SAD and other psychiatric disorders is even higher, ranging from 60 to 90% (Acarturk et al., 2008; Fehm et al., 2008; Ruscio et al., 2008). An epidemiological study using data from an adult community sample in the United States reported that women with SAD more often fulfill criteria for co-existing internalizing disorders, while men more often have comorbid externalizing disorders (Xu et al., 2012). Among adolescents, higher risk for comorbid depression has been reported among girls (Beesdo et al., 2007). The majority of knowledge on social anxiety, its sociodemographic correlates, comorbidities, short- and long-term consequences are based on studies of individuals filling all diagnostic criteria of SAD (Fehm et al., 2008). There is an increasing tendency, however, to express social anxiety along a continuous spectrum of symptom severity, most often self-reported, that also takes into account subclinical forms of the condition (Dell’Osso et al., 2003, 2014, 2015; Fehm et al., 2008; Knappe et al., 2009; Filho et al., 2010; Crişan et al., 2016). Importantly, individuals may report high levels of social anxiety symptoms without necessarily reaching the diagnostic threshold (Rapee and Spence, 2004; Spence and Rapee, 2016). There is good evidence that individuals with subclinical social anxiety also experience functional impairment across several aspects of life and have an elevated risk of comorbid psychiatric disorders (Fehm et al., 2008; Filho et al., 2010; Crişan et al., 2016). In other words, the total number of individuals with social anxiety who experience stress and impairments likely exceeds the estimated prevalences of SAD (Jefferies and Ungar, 2020). Reflecting this issue, prevalence studies based on self-report questionnaires with diagnostic cutoffs tend to report higher prevalence rates (Inglés et al., 2010; Gren-Landell et al., 2011; Jefferies and Ungar, 2020) than studies based only on diagnostic interviews (Demir et al., 2013; Canals et al., 2019; Georgiades et al., 2019). Moreover, differences between interview- and self-report – based prevalence rates could be due to the nature of social anxiety itself, as fear of an interview situation may result in non-attendance or underreporting of symptom severity. SAD often goes untreated (Wittchen et al., 1999; Grant et al., 2005). Despite the disorder’s young debut age, an adult study found the mean age of first treatment to be 27 years (Grant et al., 2005). Some suggested explanations for the avoidance of seeking professional help include fear of social interactions and authority figures, perceiving one’s symptoms as normal personality traits, and a lack of information on how SAD symptoms could be treated (National Collaborating Centre for Mental Health, 2013). In the present study, we use population data from the social anxiety project in the school-based Young-HUNT3 study, Norway (Holmen et al., 2013), which contains information from both self-reported social anxiety symptom questionnaires, and diagnostic interviews: Anxiety Disorders Interview Schedule for DSM IV: child version (ADIS-C) (Rasmussen and Neumer, 2015). The purposes of this study were: (1) to describe the occurrence of ADIS-C screening positives, SAD cases, and self-reported social anxiety symptoms among 8,199 Norwegian adolescents aged 13–19 years who participated in the population-based Young-HUNT3 study (2006–2008); (2) to examine associations between sociodemographic correlates and different subgroups of social anxiety, namely those who screened positive (SP) on the ADIS-C, SPs who did not meet for a diagnostic interview (NMI), and diagnosed cases of SAD; and (3) to assess psychiatric comorbidities among adolescents diagnosed with SAD.

## Materials and Methods

### Sampling and Procedure

**FIGURE 1:**
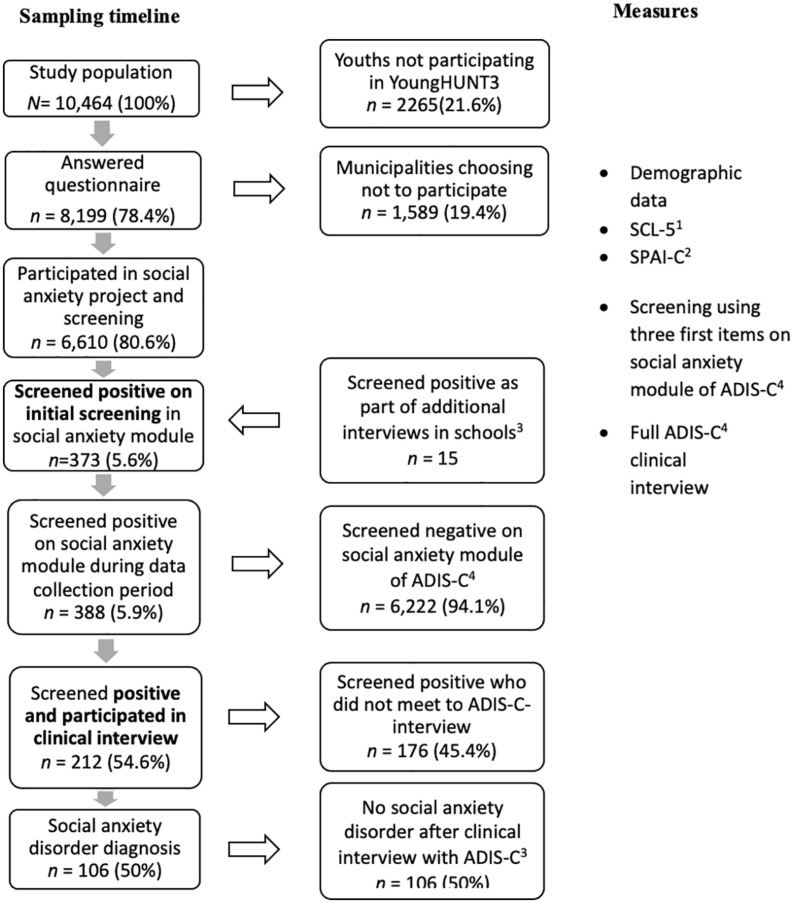
Flowchart of participants in the study. ^1^Self-reported general anxiety and depression symptoms (Derogatis et al., 1974). ^2^Self-reported social anxiety symptoms (Beidel et al., 1995). ^3^As part of ADIS-C clinical interviews in schools, a sample of 195 *presumably healthy individuals* was included in additional interviewing. Fifteen in this sample, of which seven had not previously participated in screening, as well as eight had previously screened negative, screened positive in the new round and were included as screening positive. ^4^The Anxiety Disorders Interview Schedule for DSM IV: Child Version (Rasmussen and Neumer, 2015).

Our sample included all adolescents aged 13–19 years participating in Young-HUNT3, the third wave of the Trøndelag Health Study (HUNT; 2006–2008; Holmen et al., 2013). Young-HUNT3 was a large population-based health study in which all residents of Nord Trøndelag County, Norway, aged 13–19 years were invited to participate. The county has about 127,000 inhabitants and is considered fairly representative of the Norwegian population, though it has no large cities and its education and average income levels are somewhat lower than the national average (Holmen et al., 2003). Further details regarding HUNT and the Young-HUNT study are available elsewhere (Holmen et al., 2003; Krokstad et al., 2013). Young-HUNT3 included self-report questionnaires covering a wide range of demographic, health, and behavioral factors, as well as validated instruments for social anxiety and depression symptoms. The questionnaire was administered and completed at school during school hours. Young-HUNT3 also included various health exams and clinical interviews performed approximately 1 month after the survey (Holmen et al., 2013). The social anxiety project involved an initial screening and subsequent diagnostic interviews (ADIS-C) of potential cases. The process is described in further detail below. A total of 10,464 adolescents were invited. Students absent from school on the day of the survey received the questionnaire on the day of the health exams and interviews, whereas adolescents who did not attend school either of these 2 days received the questionnaire by mail (Holmen et al., 2013). Of the adolescents invited, 8,199 completed the questionnaire (response rate = 78.4%) – 4,128 (50.4%) girls and 4,071 (49.7%) boys. The mean age was 15.9 years for both girls and boys. Two municipalities chose not to participate in the social anxiety project component, resulting in the exclusion of 1,589 adolescents in the screening process and a total of 6,610 remaining participants in the social anxiety sub-study. For details regarding the sample process, see flowchart (Figure 1).

### Measures

#### Questionnaire (*n* = 8199)

##### Descriptive variables

*Family financial situation* was measured with a question asking whether the adolescent evaluated his or her family economic situation as better than, worse than, or equal to others. *Sleep problems* were measured using two items, one related to difficulty initiating sleep (“During the last month, have you had any problems falling asleep at night?”) and one regarding early morning awakening (“During the last month, have you woken up early, and not been able to fall asleep again?”), with the response options “almost every night,” “often,” “occasionally,” and “never.” In the statistical analyses, the four alternatives were merged to create two categories: “almost every night/often” and “occasionally/never.” *Self-rated health* was assessed with the question “How is your health at the moment?” with the response options “very good,” “good,” “not so good,” and “poor.” In the statistical analyses, the alternatives were merged to create two categories: “very good/good” and “not so good/poor.” Regarding *health services*, participants were asked whether, during the previous 12 months, they had visited a general practitioner, a hospital doctor, a child healthcare clinic run by nurses, the school health services, a psychologist, a physiotherapist, a chiropractor, and/or other practitioner (naturopath, reflexologist, laying on of hands, healer, psychic, etc.) with the response options “yes” and “no” for each of the providers. *Physical activity* was measured with the item “In your leisure time, how often do you usually exercise so that you get out of breath or sweat?” with the response options “every day,” “4–6 days a week,” “2–3 times a week,” “once a week,” “less than once a week,” “less than once a month,” and “never.” Like previous Young-HUNT studies (Rangul et al., 2008; Skrove et al., 2013; Mangerud et al., 2014), the response options were categorized into three groups: “low activity” (1 day a week or less), “moderate activity” (2–3 days a week), and “high activity” (4 days a week or more). *Alcohol use* was measured with two items: – “Have you ever tried to drink alcohol?” and “Do you occasionally drink alcohol now?” Those who answered “yes” to both questions were further asked “Have you ever drunk so much alcohol that you felt intoxicated (drunk)?” and were presented with six response options: “no, never”; “yes, once”; “yes, 2–3 times”; “yes, 4–10 times”; “yes, 11–25 times”; and “yes, more than 25 times.” In accordance with established practice (Strandheim et al., 2009; Ranøyen et al., 2014), we divided answers into three categories: no, 1–10 times, and 11 times or more. *Smoking* was measured with the item “Have you ever tried to smoke?” to which participants responded with “yes” or “no.” Those who answered “yes” were further asked “Do you currently smoke?” and were presented with the response options “Yes, I smoke __ cigarettes daily”; “Yes, I smoke occasionally, but not daily”; “No, not anymore, but previously I smoked occasionally”; “No, not anymore, but previously I smoked __ cigarettes daily”; and “No, I don’t smoke.” The options were categorized into two groups based on whether the participant was a current smoker.

##### Self-reported social anxiety symptoms: SPAI-C

The Young-HUNT questionnaire included a shortened version of the originally 26-item Social Phobia and Anxiety Inventory for Children (SPAI-C; Beidel et al., 1995), which describes six symptoms of social anxiety rated on a five-point Likert scale. The SPAI-C is a DSM-IV – based self-report instrument developed by Beidel et al. (1995), which was translated into Norwegian by Aune and Hjemdal (2017). It has demonstrated adequate validity and reliability for use among adolescents (Storch et al., 2004). A mean score (Cronbach’s alpha = 0.84) across the six SPAI-C items was calculated for each of the subgroups of social anxiety, with higher scores indicating elevated symptom levels.

##### Anxiety and depression symptoms (SCL-5)

General symptoms of anxiety and depression in the previous 2 weeks were assessed using a shortened five-item version of the 25-item Symptom Checklist (SCL; Derogatis et al., 1974), which performs similarly to the full version (Strand et al., 2003). A mean score was calculated (Cronbach’s alpha = 0.83) across the five items, with higher scores indicating elevated symptom levels.

#### Social Anxiety Disorder Screening and Clinical Interview (*n* = 6,610 and 212)

##### Anxiety Disorders Interview Schedule for DSM IV: Child Version

The Anxiety Disorders Interview Schedule for DSM IV: Child Version is a semi-structured interview used to diagnose anxiety disorders and other mental disorders in children and adolescents, according to the DSM-IV criteria (American Psychiatric Association, 2000; Rasmussen and Neumer, 2015). In the present study, the interview modules for SAD, generalized anxiety disorder (GAD), separation anxiety disorder (SEP), specific phobias (SPH), obsessive-compulsive disorder (OCD), post-traumatic stress disorder (PTSD), dysthymia, and depression were used. For more convenient administration and coding, the modules were slightly shortened. In addition, questions regarding symptoms of substance abuse were asked, yet a diagnostic evaluation of substance abuse cannot be set based on the ADIS-C interview alone (Rasmussen and Neumer, 2015). The original version of ADIS-C has shown promising reliability (Lyneham et al., 2007), whereas research on psychometric properties of the Norwegian version is limited (Rasmussen and Neumer, 2015). However, the instrument is widely used in specialist health service, and items largely resemble the diagnostic criteria described in DSM-IV. It is highly recommended that it is used/applied only by trained clinicians with knowledge to the instrument and the diagnostic criteria (Rasmussen and Neumer, 2015). All participants (*n* = 6,6610) were asked the following three social anxiety items from the ADIS-C (yes/no): “When you are with others, at school, in restaurants or at parties, do you ever feel that people might think that something you do is stupid or dumb?”; “When you are with other people at school, restaurants, or parties, do you think that people might laugh at you?”; and “When you are in these situations with others (school, restaurants, and parties), do you worry that you might do something that will make you feel ashamed or embarrassed?” Individuals who answered yes to one or more questions were considered SP (*n* = 388) and invited to participate in a complete ADIS-C interview performed by specially trained psychiatric nurses. Those who answered no to all three questions were considered screening negative (SN; *n* = 6,222).

### Groups

The initial screening phase yielded 373 SP individuals (5.6%), who were subsequently invited to complete an ADIS-C interview. In another part of the overall project, a sample of presumably healthy individuals (*n* = 195) was also interviewed with ADIS-C. Of these, 15 SP for SAD. Making it a total of 388 (5.9%) that SP at one time during Young-HUNT3. Among these, 212 participated (response rate = 54.6%), of whom 106 met the criteria for SAD. A total of 176 SPs (45.4%) did not participate.

In the statistical analyses, the study sample was divided into subgroups:

(1) ADIS-C SN (*n* = 6,222; answered no to all three screening questions).
(2) ADIS-C SP (*n* = 388; answered yes to one or more screening questions).
(3) ADIS-C SP that did not meet to interview (NMI; *n* = 176).
(4) ADIS-C SP diagnosed with SAD, indicated by ADIS-C interview with trained nurse (*n* = 106).

Group 3 and 4 represent subgroups of the SPs.

### Statistics

Stata version 15.1 (StataCorp, 2017) was used for data management and statistical analyses. First, a descriptive analysis was performed for the four study groups regarding distributions of sex, age, perception of family economic situation, self-reported social anxiety symptoms (based on SPAI-C), general anxiety and depression symptoms (based on SCL-5), sleep problems, self-rated health, professional healthcare seeking in last 12 months, physical activity, number of alcohol intoxications, and smoking behavior. Further, logistic regressions were performed to estimate age- and sex-adjusted odds ratios (ORs) between these sociodemographic variables and the different social anxiety subgroups. Third, a sub-analysis of psychiatric comorbidity among the individuals diagnosed with SAD was performed, with each comorbid condition listed separately. The sum of individuals with comorbid disorders exceeded the total number of SAD cases as a result of some participants meeting the criteria for more than one comorbid condition.

### Screening Negatives (*n* = 6,222) as Reference Group

When comparing the SAD sample (*n* = 106) to SNs as a reference group, SPs without a SAD diagnosis were coded as missing (*n* = 282). Additional analyses were performed wherein SPs without a SAD diagnose were included in the reference group, yet the results largely remained the same.

### Ethics

Participation in the study was voluntary. Participants signed a letter of informed consent prior to participation. For students under the age of 16, written consent from a parent was necessary (Holmen et al., 2013). The research protocol was approved by the Regional Committee for Medical and Health Research Ethics.

## Results

### Sociodemographic Characteristics

Of the 6,610 adolescents, 6,222 (94.1%) were ADIS-C SN and 388 (5.9%) were ADIS-C SP. After invitation, 212 (54.6% of adolescents in the SP group) participated in the interview. Of these, 106 (50%) fulfilled the diagnostic criteria for SAD. A total of 176 SPs (45.4%) did not participate in the clinical interview (NMI). Table 1 shows the descriptive characteristics for the four subgroups (SP, SN, NMI, and SAD).

**TABLE 1:** Descriptive characteristics of adolescents in Young-HUNT3 categorized/identified as Anxiety Disorders Interview Schedule for DSM IV: child version (ADIS-C) screening negative, ADIS-C screening positive, screening positive not met to interview, and as diagnosed social anxiety disorder (SAD) cases.

	SAD (ADIS-C) screening neg (*n* = 6,222)	SAD (ADIS-C) screening pos (*n* = 388)		
	
		All screening positives (*n* = 388)	Screening positives that did not meet to interview (*n* = 176)	Screening positives met to interview and diagnosed with SAD (*n* = 106)
**Sex, *n* (%)**				
Girls	3,063 (49.23)	267 (68.81)	120 (68.18)	85 (80.19)
Boys	3,159 (50.77)	121 (31.19)	56 (31.82)	21 (19.81)
Age mean (SD)	15.97 (1.70)	16.12 (1.90)	16.47 (2.13)	15.74 (1.63)
**Age distribution, *n* (%)**				
13–15 years	3,176 (51.04)	195 (50.26)	76 (43.18)	62 (58.49)
≥16 years	3,046 (48.96)	193 (49.74)	100 (56.82)	44 (41.51)
**Subjective family economy^3^, *n* (%)**				
Worse than others	497 (8.46)	57 (15.92)	29 (18.13)	17 (17.35)
Mean all social anxiety items (SPAI-C) (sd)	1.86 (0.01)	2.82 (0.05)	2.86 (0.07)	3.04 (0.09)
Girls (mean all social anxiety items)	2.00 (0.67)	2.91 (0.85)	2.96 (0.85)	3.07 (0.87)
Boys (mean all social anxiety items)	1.71 (0.64)	2.63 (0.90)	2.65 (0.84)	2.91 (1.11)
Mean anxiety and depression items (SCL-5) (SD)	1.47 (0.01)	2.01 (0.04)	2.05 (0.06)	2.11 (0.07)
**Difficulties falling asleep, *n* (%)**				
Almost every night/often	890 (14.83)	115 (31.08)	54 (32.53)	33 (33.00)
**Early morning awakening, *n* (%)**				
Almost every night/often	342 (5.72)	55 (14.99)	30 (18.29)	12 (12.12)
**Self-rated health, *n* (%)**				
Very good/good	5,536 (90.19)	293 (77.72)	132 (77.65)	81 (78.64)
Not very good/poor	602 (9.81)	84 (22.28)	38 (22.35)	22 (21.36)
**Help-seeking, *n* (%)**				
Psychologist	252 (4.39)	55 (16.18)	30 (19.87)	18 (18.75)
School health service	1,249 (21.79)	91 (26.84)	36 (23.84)	34 (35.79)
Doctor at hospital	1,719 (29.86)	130 (37.68)	55 (35.95)	42 (43.75)
All	3,660 (67.48)	247 (77.43)	108 (78.83)	80 (88.89)
**Physical activity, *n* (%)**				
High	2,555 (41.59)	89 (23.61)	40 (23.81)	18 (16.98)
Moderate	2,139 (34.82)	149 (39.52)	59 (35.12)	51 (48.11)
Low	1,449 (23.59)	139 (36.87)	69 (41.07)	37 (34.91)
**Alcohol intoxications, *n* (%)**				
Never	3,087 (49.98)	217 (56.66)	94 (54.34)	65 (61.90)
1–10 times	1,590 (25.74)	97 (25.33)	35 (20.23)	30 (28.57)
>10 times	1,499 (24.27)	69 (18.02)	44 (25.43)	10 (9.52)
**Smoking, *n* (%)**				
Current smoker	877 (14.37)	63 (16.54)	31 (18.02)	17 (16.19)

*Note. Missing values ranged between 0.9% (alcohol intoxications) and 14.4% (all help). Regarding the SPAI-C questions, missing values ranged between 2.8 and 3.2% across the six items, and the summed mean SPAI-C score missed values for 4.4% of the participants (19 (4.9%) of the screening positives, and 4 (3.8%) of the SAD individuals). For SCL-5, missing values ranged between 2.6 and 2.8% across the five items, and the summed total mean score missed values for 3.5% (16 (4.1%) of the screening positives, 2 (1.9%) of the SAD individuals).*

A larger proportion of girls than boys SP for and were diagnosed with SAD (SP: 69%; SAD: 80%). Within the interview group (*n* = 212), a higher proportion of girls than boys fulfilled the criteria for SAD (girls: 58%; boys: 32%). Average self-reported social anxiety symptom levels increased across the subgroups, from 1.86 points in the SN group to 3.04 in the SAD group. Similarly, mean SCL-5 values were 1.47 (SN), 2.01 (SP), 2.05 (NMI), and 2.11 (SAD). Compared to the SN group, higher proportions of adolescents in the three SP subgroups reported their family economic situation as “worse than others” (SN: 8.5%; SP groups: 15.9–18.1%); self-rated their health as “not so good/poor” (SN: 9.8%; SP: 21.4–22.4%); had visited a healthcare professional in the previous 12 months (SN: 67.5%; SP: 77.4–88.9%); and had sleep problems often or almost every night – both difficulty falling asleep (SN: 14.8%; SP: 31.1–33.0%) and early morning awakening (SN: 5.7%; SP: 12.1–18.3%). Further, a lower proportion of the SP subgroups reported high levels of physical activity (SN: 41.6%; SP: 17.0–23.8%), and a lower proportion of the SAD group had experienced 10 or more alcohol intoxications compared to the other groups (SAD: 9.5%; SN: 24.3%; SP: 18.0%; NMI: 25.4%). For remaining descriptive information about the four subgroups, see Table 1.

Table 2 shows the associations (ORs and 95% confidence intervals [CIs]) between sociodemographic variables and the different outcome subgroups. Adjusted for age, girls had twice the odds of being both SP (OR: 2.3, 95% CI: [1.82, 2.84]) and NMI (OR: 2.2, 95% CI: [1.60, 3.04]) and four times the odds of having SAD (OR: 4.2, 95% CI: [2.59, 6.76]) compared to boys. Adjusted for sex, there was no difference in SP across age categories. Adjusted for age and sex, reporting a worse family economic situation was associated with a doubled odds of being SP (OR: 1.93, 95% CI: [1.42, 2.62]) and NMI (OR: 2.20, 95% CI: [1.44, 3.36]) and of having SAD (OR: 2.04, 95% CI: [1.19, 3.50]) in all outcome subgroups compared to the group reporting an equal family economic situation. A one-unit increase in SPAI-C score was associated with four times higher odds of being SP (OR: 4.17, 95% CI: [3.64, 4.78]) and NMI (OR: 4.16, 95% CI: [3.47, 4.99]) and five times higher odds of having SAD (OR: 4.99, 95% CI: [3.98, 6.26]). A one-unit increase in SCL-5 score was associated with 3.3 to 3.5 times higher odds of being in an SP subgroup. Adjusted for age and sex, adolescents who reported sleeping problems (both difficulty falling asleep and early morning awakening) had two to three times the odds of being in an SP subgroup compared to those without sleeping problems. Those reporting poor self-rated health had 2.5 times higher odds of being in an SP subgroup compared to those reporting good health. Having visited any healthcare professional in the last year was associated with higher odds of being SP (OR: 1.44, 95% CI: [1.09, 1.90]) and having SAD (OR: 3.41, 95% CI: [1.75, 6.64]). Having visited a psychologist in the last year was additionally associated with 3.8–4.7 times higher odds of SP compared to those who had not visited a psychologist. Reporting 10 or more alcohol intoxications was associated with lower odds of having SAD (OR: 0.28, 95% CI: [0.13, 0.60]) compared to not having tried alcohol or never having experienced any alcohol intoxication. Finally, reporting moderate or low levels of physical activity was associated with increased odds of being in SP subgroups, compared to reporting high levels of physical activity.

**TABLE 2:** Age- and sex adjusted associations (odds ratio and 95% confidence interval) between sociodemographic and health-related variables and the different subgroups of social anxiety.

	SAD (ADIS-C) screening pos (*n* = 388)					
	All screening positives (*n* = 388)		Screening positives that did not meet to interview (*n* = 176)		Screening positives met to interview and diagnosed with SAD (*n* = 106)	
			
	OR	95% CI	OR	95% CI	OR	95% CI
Sex^1^						
Boys	1	Ref	1		1	
Girls	2.28	1.82–2.84	2.20	1.60–3.04	4.18	2.59–6.76
Age distribution^2^						
13–15 years	1		1	1		
≥16 years	1.02	0.83–1.26	1.36	1.01–1.84	0.73	0.50–1.08
Family economy^3^						
Equal	1		1		1	
Worse	1.93	1.42–2.62	2.20	1.44–3.36	2.04	1.19–3.50
Better	0.88	0.65–1.20	0.84	0.53–1.35	0.51	0.24–1.06
SPAI-C^3^	4.17	3.64–4.78	4.16	3.47–4.99	4.99	3.98–6.26
SCL-5^3^	3.25	2.79–3.79	3.36	2.72–4.15	3.49	2.70–4.50
Difficulties falling asleep^3^						
Occasionally/never	1		1		1	
Almost every night/often	2.33	1.85–2.95	2.49	1.78–3.48	2.40	1.57–3.68
Early morning awakening^3^						
Occasionally/never		1		1		1
Almost every night/often	2.67	1.96–3.64	3.38	2.24–5.12	2.03	1.09–3.76
Self-rated health^3^						
Very good/good	1		1	1		
Not very good/poor	2.52	1.94–3.26	2.46	1.69–3.57	2.44	1.51–3.97
Help seeking^3^						
No help seeking	1		1	1		
Psychologist	3.80	2.76–5.24	4.67	3.05–7.15	4.65	2.72–7.98
School health service	1.15	0.89–1.47	0.97	0.66–1.43	1.65	1.07–2.53
Doctor at hospital	1.35	1.08–1.69	1.23	0.88–1.72	1.72	1.14–2.59
Physical activity^3^						
High	1		1		1	
Moderate	1.87	1.43–2.45	1.65	1.10–2.49	3.06	1.78–5.26
Low	2.63	1.99–3.46	2.82	1.90–4.21	3.50	1.98–6.20
Alcohol intoxications^3^						
Never	1		1	1		
1–10 times	0.72	0.54–0.94	0.53	0.34–0.81	0.79	0.49–1.28
>10 times	0.51	0.37–0.71	0.63	0.40–0.98	0.28	0.13–0.60
Smoking^3^						
Never/previous smoker	1		1		1	
Current smoker	1.14	0.85–1.52	1.16	0.77–1.74	1.23	0.71–2.13

*^1^Adjusted only for age.^2^Adjusted only for sex.^3^Adjusted for age and sex.*

## Comorbidity Among SAD Individuals

In total, 75 (72.8%) of the SAD cases – 62 (75.6%) girls and 13 (61.9%) boys – had one or more comorbid condition(s). GAD was the most frequent, diagnosed in 62 (58.5%) of SAD cases, followed by SPH, diagnosed in 27 cases (25.7%). Other anxiety disorders were diagnosed in 74 (71.2%) of the SAD cases whereas depression was diagnosed in 24 (23.1%), and dysthymia in 13 (13.1%). The least frequent comorbid conditions was PTSD diagnosed in 11 (10.8%), SEP diagnosed in six (5.9%), and likely substance abuse, which was detected in only four girls. 28 SAD cases (27.2%) had no comorbid psychiatric conditions. Percentages for the remaining comorbid psychiatric disorders are listed in Table 3.

**TABLE 3:** Prevalence of comorbidity with other psychiatric disorders among Young-HUNT3 adolescents diagnosed with SAD (*n* = 106).

	Total (%)	Girls *n* (%)	Boys (%)
SAD, single condition	28 (27.18)	20 (24.39)	8 (38.10)
Any comorbid condition	75 (72.82)	62 (75.61)	13 (61.90)
Other anxiety disorders^1^	74 (71.15)	61 (73.49)	13 (61.90)
Generalized anxiety disorder (GAD)	62 (58.49)	50 (58.82)	12 (57.14)
Specific phobias (SPH)	27 (25.71)	23 (27.38)	4 (19.05)
Obsessive compulsive disorder (OCD)	24 (23.08)	21 (25.30)	3 (14.29)
Depression	24 (23.08)	23 (27.71)	1 (4.76)
Dysthymia	13 (13.13)	13 (16.67)	0 (0.00)
Post-traumatic stress disorder (PTSD)	11 (10.78)	10 (12.20)	1 (5.00)
Separation anxiety disorder (SEP)	6 (5.94)	5 (6.10)	1 (5.26)
Possibility of substance abuse	4 (3.81)	4 (4.76)	0 (0.00)

*^1^One or several of the following: GAD, SPH, OCD, and PTSD or SEP.Note. Missing values for SAD cases for the following comorbid diagnoses: dysthymia (n = 7), depression (n = 2), SEP (n = 5), PTSD (n = 4), OCD (n = 2), substance abuse (n = 1), and SPH (n = 1).*

## Discussion

In this large population-based study of more than 6,000 Norwegian adolescents, we found that higher mean scores of self-reported social anxiety symptoms, poor self-rated health, sleep problems, poor family economic situation, low physical activity, and professional help-seeking in the previous year all were associated with higher odds of being in SP subgroups. Girls were overrepresented in both the SP and SAD groups, and more than two-thirds of adolescents diagnosed with SAD had one or more comorbid psychiatric disorders. On the whole, our results are comparable with previous studies of adolescent social anxiety in terms of gender differences (Asher et al., 2017), comorbidity (Wittchen et al., 1999; Ranta et al., 2009; Spence et al., 2018; Canals et al., 2019), and sleep problems (Brown et al., 2018).

### Sociodemographic Characteristics

A total of 106 adolescents had a indicated diagnosis of SAD, representing 1.6% of the total population participating in the Young-HUNT3 social anxiety project (*n* = 6,610). This is considerably lower than prevalence rates described in most adolescent community diagnostic studies in the Western world covering the same age group, which have reported between 3.2 and 8.2% prevalence (Ranta et al., 2009; Kessler et al., 2012; Spence et al., 2018; Georgiades et al., 2019). The most likely explanation for the low SAD occurrence in our study is the low response rate (54.6%) among the SP individuals invited to diagnostic interviews. In addition, it is important to recognize that differences in diagnostic methods (Costello, 2015) as well as cultural differences (Hofmann et al., 2010) are likely contributing factors to these findings. In addition, the use of different reference periods (prevalence over 30 days, 6 months, 12 months, and/or lifetime) could explain divergent findings in the occurrence of SAD across samples and settings. Our findings of female predominance are in accordance with several previous community studies of social anxiety in adolescents (Wittchen et al., 1999; Kessler et al., 2012; Canals et al., 2019; Georgiades et al., 2019). In a recent review of gender differences in SAD, it was concluded that women were more likely to fill the diagnostic criteria for SAD (Asher et al., 2017). In the same review, Asher et al. (2017) discuss the role of reporting bias in social anxiety; whether or not boys tend to underreport their symptoms on purpose to reduce the inconsistency between how they feel and how they want to appear in order to fit gender roles. One could hypothesize that the interview situation itself could have made boys more hesitant to reveal their symptoms. However, little empirical evidence exists for this type of bias (Asher et al., 2017). In fact, results from an adult study of individuals with SAD may indicate the opposite: Women with lifetime SAD were more likely to fear professional situations such as being interviewed and talking to authority figures more than men (Xu et al., 2012). Regardless, internalizing mental disorders are more common among girls (Herpertz-Dahlmann et al., 2013) and therefore perhaps also more socially acceptable. There may also exist gender differences in willingness to report on different fears, which could affect the gender distribution (Xu et al., 2012). Moreover, there were also gender differences in self-reports. Girls across all three SP subgroups reported higher mean values of self-reported social anxiety symptoms. Our findings contrast those of Sanna et al. (2009), who did not find gender differences in self-reported social anxiety symptoms among Finnish students aged 8–16 years using the 26-item SPAI-C. A Swedish study, however, found a higher prevalence of SAD among female high school students using self-reports with a diagnostic cutoff (Gren-Landell et al., 2011). Intriguingly, a Turkish study of 1,713 students aged 10–16 years reported higher social anxiety symptoms among boys (Cakin Memik et al., 2010). The ages of participants as well as methodological, cultural, and social differences may explain these discrepancies. Participants who reported that their family economic situation was worse than others had greater odds of being in the SP and SAD groups compared with those who reported having an equal family economic situation. This lends further support to a previously documented association between subjective perception of family economic situation and social anxiety (Wittchen et al., 1999; Ranøyen et al., 2014). Evaluation of the family’s economic situation could be interpreted as a form of subjective socioeconomic status (SES), which has been shown to be associated with health outcomes, especially mental health outcomes (Quon and McGrath, 2014). Since one of the characteristics of social anxiety is the fear of being negatively evaluated by others (American Psychiatric Association, 2000), we believe that subjective SES is an appropriate measure for use among adolescents in our study. Our study lacks objective information on parental SES. However, using parental SES to describe adolescent SES could be disadvantageous (Glendinning et al., 1992), since parental SES and adolescents’ perception of their own place in the social hierarchy are not necessarily correlated (Quon and McGrath, 2014). On the other hand, children and adolescent studies have reported associations between objective (although self-reported) low family SES and SAD (Canals et al., 2019). A positive association was found between help-seeking in the last 12 months and being SP. Reporting having visited a psychologist was associated with a four times higher odds of being SP. This association is in line with Spence et al. (2018) study of Australian children and adolescents with SAD where 73% had received help within last 12 months. However, it contrasts several retrospective adult studies reporting that, despite the early debut of the condition, only a small portion contact the healthcare system (Fehm et al., 2005; Grant et al., 2005), and most do so after living with their symptoms for many years (Fehm et al., 2005). Due to evidence that individuals with SAD are more likely to seek help because they are bothered by symptoms of comorbid disorders rather than symptoms of SAD (Ranta et al., 2009), as well as the fact that 70% of individuals diagnosed with SAD in our study had one or more comorbid disorders, symptoms from comorbid disorders could naturally also represent reasons to contact the healthcare system. There was a positive association between sleep problems (both insomnia symptoms and early morning awakening) and being in an SP subgroup. This is in line with previous literature reporting that sleep problems are prevalent in children and adolescents with anxiety disorders (Brown et al., 2018). There was a negative association between high number of alcohol intoxications and having SAD, compared with those who had never tried alcohol or had never experienced alcohol intoxications. This is most likely explained by the low response rate among SP meeting to diagnostic interview (=54.6%), due to the higher percentage of NMI having experienced >10 alcohol intoxications (Table 1), compared to the other SP subgroups. Among adults with SAD, alcohol is often used to reduce social anxiety symptoms (Carrigan and Randall, 2003), and alcohol use disorders typically co-occurs with SAD (Morris et al., 2005). Also among adolescents, an association between self-reported social anxiety symptoms and coping motives for drinking alcohol has been reported (Blumenthal et al., 2010). However, due to the low response rate our results are not comparable to the abovementioned studies. Determining whether social anxiety is best described as a spectrum of symptoms or categorically, in the form of a diagnosis or no diagnosis, is challenging (Hyett and McEvoy, 2018). Therefore, we chose to include both established measures of symptoms and diagnostic assessment. However, since one of the main characteristics of social anxiety is avoidance of social situations (American Psychiatric Association, 2000), the diagnostic interview could represent an obstacle and may partly explain why only 54.6% of the SP group participated in the diagnostic interview. Considering this, along with the fact that the NMI group did not differ notably from the rest of the SP group in terms of sociodemographic characteristics or self-reported symptoms, one could argue that the use of self-reports and ADIS-C screening questions could actually be sufficient to identify relevant population based cohorts of adolescents at risk of social anxiety.

### Comorbidity

In total, 75 (72.8%) of the SAD individuals in our study fulfilled the criteria for one or more comorbid conditions. High mental comorbidity is in line with several previous community based child and adolescent studies of SAD (Wittchen et al., 1999; Ranta et al., 2009; Spence et al., 2018; Canals et al., 2019; Mohammadi et al., 2020) and lends support to the idea that “comorbidity seems to be the rule rather than the exception” among adolescents with SAD (Fehm et al., 2005, p. 456). GAD was the most prevalent (58.5%) comorbid condition, followed by SPH (25.7%). High comorbidity between SAD and GAD has also been reported in several previous studies. Although they used parent reports and only assessed SAD, GAD, and SEP, Spence et al. (2018) also reported GAD as the most prevalent comorbid condition, present in 38.4% of Australian adolescents aged 12–17 years diagnosed with SAD. In addition, Mohammadi et al. (2020) reported comorbid GAD in 26% of 585 Iranian SAD cases aged 6–18 years. In a Spanish study of children and adolescents, Canals et al. (2019) reported GAD as the second most prevalent comorbid condition (39.1%) after SPH (43.5%), and Garcia-Lopez et al. (2016) recommended screening for both GAD and specific phobia when assessing adolescents with SAD. It has also been questioned, by Maj (2005), if psychiatric “comorbidity” is an incorrect term, because it is unclear whether it is actually co-occurrence of several diagnoses, or whether it is just an artifact due to the diagnostic systems that do not precisely account for numerous manifestations of a single condition. Only six (5.9%) of the individuals with SAD in our study fulfilled the criteria for SEP. In a review regarding anxiety disorders, Beesdo-Baum and Knappe (2012) concluded that SEP tend to be more prevalent among children compared to adolescents. The close link between SAD and depression found in our sample (23.1%) is in agreement with previous findings in adolescent community studies using diagnostic criteria (Wittchen et al., 1999; Ranta et al., 2009; Spence et al., 2018; Canals et al., 2019). Co-existing depression and SAD in adolescence is known to generate a more severe course of the depressive disorder. Moreover, the presence of SAD in adolescence increases the risk of subsequent affective disorders (Stein et al., 2001). Only four (3.8%) of the individuals diagnosed with SAD – all of them girls – met the screening criteria for possibility of substance abuse, representing the least frequent comorbid condition. Due to this very small number (*n* = 4) along with small number of boys in NMI group (*n* = 56) and SAD group (*n* = 21), careful interpretation of the results is necessary. Wu et al. (2010) study of adolescents with SAD found a negative association between social phobia and drug use in girls only. However, due to the small numbers in our study, the results are not comparable. Only 28 (27.2%) of individuals with SAD did not fill criteria for any additional mental disorders measured by the ADIS-C.

## Strengths and Limitations

The main strength of this study is the large number of participants, covering all adolescents in the Nord Trøndelag county of Norway, as well as the high response rate on the questionnaire (=78.4%). In addition, the data on social anxiety were based on both self-reports, and diagnostic interviews. Furthermore, population studies are advantageous when studying social anxiety, as they can capture mentally healthy, anxious but not previously diagnosed, and already diagnosed individuals. Regarding limitations, the study may be subject to selection bias. First, due to the nature of the condition, severe sufferers of SAD may have been absent from school on the day of the study. Second, only 54.6% of the SP group participated in the ADIS-C interview. Further, the SAD group consisted of only 106 individuals, and therefore careful interpretation of the results is warranted. The ADIS interview is considered gold standard in diagnosing anxiety disorders among children and adolescents aged 7–17 years (Silverman and Ollendick, 2005). However, the interview consists originally of both a child *and* a parent version (ADIS-C/P) (Rasmussen and Neumer, 2015), yet in the current study only the child version was included. The inter-rater reliability of ADIS has shown to be high for the anxiety disorders, and acceptable for the common comorbid disorders, the latter especially when including parent information (Lyneham et al., 2007). Next, although the ADIS-C interviews were performed by nurses who were specially trained, one cannot omit the possibility of achieving other results if the interviews were conducted by experienced physicians or psychologists. When it comes to seeking professional help, our study did not reveal information what individuals contacted the healthcare system for. Such contacts may be made for somatic as well as psychiatric reasons. Lastly, when interpreting the results from SPs, it is important to recognize that this group included 106 SAD individuals, which presumably increased the rate of symptoms in the group.

## Conclusion

The purpose of this study was to report the occurrence of social anxiety as well as its sociodemographic correlates and mental comorbidities among adolescents aged 13–19 who participated in Young-HUNT3 (2006–2008). In total, 388 (5.9%) adolescents SP for SAD, and a complete ADIS-C interview indicated a diagnosis in 106 adolescents. SAD was four times more common in girls than boys. A total of 72.8% of adolescents with SAD had one or more comorbid psychiatric disorders, with GAD the most frequent (58.5%). Reports of poor self-rated health, sleep problems, poor family economic situation, low physical activity, and having sought professional help within the last year were associated with higher odds of being SP. The ADIS-C SPs that did not meet for a diagnostic interview did not differ markedly from the rest of the SP group in terms of these socio-demographic characteristics. Considering this, in addition to the fact that individuals with social anxiety may fear interview situations, the use of self-reports and the ADIS-C screening questions may be sufficient to identify epidemiologically relevant cohorts of adolescents at risk of social anxiety. Due to the high resource demands in studies using diagnostic interviews, this could have valuable implications for future epidemiological social anxiety research.

## Data Availability Statement

The Trøndelag Health Study (HUNT) has invited persons aged 13–100 years to four surveys between 1984 and 2019. Comprehensive data from more than 140,000 persons having participated at least once and biological material from 78,000 persons are collected. The data are stored in HUNT databank and biological material in HUNT biobank. HUNT Research Centre has permission from the Norwegian Data Inspectorate to store and handle these data. The key identification in the data base is the personal identification number given to all Norwegians at birth or immigration, whilst de-identified data are sent to researchers upon approval of a research protocol by the Regional Ethical Committee and HUNT Research Centre. To protect participants’ privacy, HUNT Research Centre aims to limit storage of data outside HUNT databank, and cannot deposit data in open repositories. HUNT databank has precise information on all data exported to different projects and are able to reproduce these on request. There are no restrictions regarding data export given approval of applications to HUNT Research Centre. For more information see: http://www.ntnu.edu/hunt/data. Requests to access the datasets should be directed to http://www.ntnu.edu/hunt/data.

## Ethics Statement

The studies involving human participants were reviewed and approved by Regional committee for medical and health research ethics. Written informed consent to participate in this study was provided by the participants’ legal guardian/next of kin.

## Author Contributions

All authors participated in planning the outlines of the study, including strategy for data usage, and analysis. IJ conducted the analysis with assistance from JV and ES. IJ wrote the outlines of the manuscript, with input and support from all authors, did the majority of work on all aspects of writing introduction, methodology, results and discussion, with support, and contribution from all authors.

## Conflict of Interest

The authors declare that the research was conducted in the absence of any commercial or financial relationships that could be construed as a potential conflict of interest.
