# Corrigendum: Sociodemographic correlates and mental health comorbidities in adolescents with social anxiety: the Young-HUNT3 study, Norway

**DOI:** 10.3389/fpsyg.2023.1163212

**Published:** 2023-05-18

**Authors:** Ingunn Jystad, Ottar Bjerkeset, Tommy Haugan, Erik R. Sund, Jonas Vaag

**Affiliations:** ^1^Faculty of Nursing and Health Science, Nord University, Levanger, Norway; ^2^Department of Public Health and Nursing, Faculty of Medicine and Health Science, Norwegian University of Science and Technology, Trondheim, Norway; ^3^Department of Mental Health, Norwegian University of Science and Technology, Trondheim, Norway; ^4^Department of Public Health and Nursing, HUNT Research Centre, Norwegian University of Science and Technology, Trondheim, Norway; ^5^Levanger Hospital, Nord-Trøndelag Hospital Trust, Levanger, Norway; ^6^Department of Psychology, Faculty of Social and Educational Sciences, Norwegian University of Science and Technology, Trondheim, Norway

**Keywords:** social anxiety disorder, adolescence, ADIS-C, self-report, sociodemographics, comorbidity, HUNT–study

In the published article, there was an error in Table 1 as published. In the rows, “Mean all social anxiety items (SPAI-C) (SD)” and “Mean anxiety and depression items (SCL-5) (SD)” the mean values for SPAI-C and SCL-5 were erroneously reported with standard errors (SE) instead of standard deviations (SD). The corrected Table 1 appears below.

**Table 1:** Descriptive characteristics of adolescents in Young-HUNT3 categorized/identified as Anxiety Disorders Interview Schedule for DSM IV: child version (ADIS-C) screening negative, ADIS-C screening positive, screening positive not met to interview, and as diagnosed social anxiety disorder (SAD) cases.

	**SAD (ADIS-C) Screening neg (*n* = 6,222)**	**SAD (ADIS-C) screening pos (*n* = 388)**		
		**All screening positives**	**Screening positives that did not meet to interview**	**Screening positives met to interview and diagnosed with SAD**
		**(*****n***=**388)**	**(*****n***=**176)**	**(*****n***=**106)**
**Sex** ***n*** **(%)**				
Girls	3063 (49.23)	267 (68.81)	120 (68.18)	85 (80.19)
Boys	3159 (50.77)	121 (31.19)	56 (31.82)	21 (19.81)
**Age mean (SD)**	15.97 (1.70)	16.12 (1.90)	16.47 (2.13)	15.74 (1.63)
**Age distribution** ***n*** **(%)**				
13–15 years	3176 (51.04)	195 (50.26)	76 (43.18)	62 (58.49)
≥16 years	3046 (48.96)	193 (49.74)	100 (56.82)	44 (41.51)
**Subjective family economy**^**3**^ ***n*** **(%)**				
Worse than others	497 (8.46)	57 (15.92)	29 (18.13)	17 (17.35)
**Mean all social anxiety items (SPAI-C) (SD)**	1.86 (0.67)	2.82 (0.87)	2.86 (0.86)	3.04 (0.92)
Girls (mean all social anxiety items)	2.00 (0.67)	2.91 (0.85)	2.96 (0.85)	3.07 (0.87)
Boys (mean all social anxiety items)	1.71 (0.64)	2.63 (0.90)	2.65 (0.84)	2.91 (1.11)
**Mean anxiety and depression items (SCL-5) (SD)**	1.47 (0.52)	2.01 (0.72)	2.05 (0.73)	2.11 (0.74)
**Difficulties falling asleep** ***n*** **(%)**				
Almost every night/often	890 (14.83)	115 (31.08)	54 (32.53)	33 (33.00)
**Early morning awakening** ***n*** **(%)**				
Almost every night/often	342 (5.72)	55 (14.99)	30 (18.29)	12 (12.12)
**Self-rated health n (%)**				
Very good/good	5536 (90.19)	293 (77.72)	132 (77.65)	81 (78.64***)***
Not very good/poor	602 (9.81)	84 (22.28)	38 (22.35)	22 (21.36)
**Help-seeking** ***n*** **(%)**				
Psychologist	252 (4.39)	55 (16.18)	30 (19.87)	18 (18.75)
School health service	1249 (21.79)	91 (26.84)	36 (23.84)	34 (35.79)
Doctor at hospital	1719 (29.86)	130 (37.68)	55 (35.95)	42 (43.75)
All	3660 (67.48)	247 (77.43)	108 (78.83)	80 (88.89)
**Physical activity** ***n*** **(%)**				
High	2555 (41.59)	89 (23.61)	40 (23.81)	18 (16.98)
Moderate	2139 (34.82)	149 (39.52)	59 (35.12)	51 (48.11)
Low	1449 (23.59)	139 (36.87)	69 (41.07)	37 (34.91)
**Alcohol intoxications n (%)**				
Never	3087 (49.98)	217 (56.66)	94 (54.34)	65 (61.90)
1–10 times	1590 (25.74)	97 (25.33)	35 (20.23)	30 (28.57)
>10 times	1499 (24.27)	69 (18.02)	44 (25.43)	10 (9.52)
**Smoking** ***n*** **(%)**				
Current smoker	877 (14.37)	63 (16.54)	31 (18.02)	17 (16.19)

Missing values ranged between 0.9% (alcohol intoxications) and 14.4% (all help).

Regarding the SPAI-C questions, missing values ranged between 2.8 and 3.2% across the six items, and the summed mean SPAI-C score missed values for 4.4% of the participants (19 (4.9%) of the screening positives, and 4 (3.8%) of the SAD individuals). For SCL-5, missing values ranged between 2.6 and 2.8% across the five items, and the summed total mean score missed values for 3.5% (16 (4.1%) of the screening positives, 2 (1.9%) of the SAD individuals).

In addition, there was also an error in Table 2 as published. The row “All” in the section “Help Seeking” was erroneously excluded. The corrected Table 2 appears below.

**Table 2:** Age- and sex adjusted associations (odds ratio and 95% confidence interval) between sociodemographic and health-related variables and the different subgroups of social anxiety.

			**SAD (ADIS-C) screening pos (n**=**388)**			
	**All screening positives (*****n*** = **388)**		**Screening positives that did not meet to interview (*****n*** = **176)**		**Screening positives met to interview and diagnosed with SAD (*****n*** = **106)**	
	**OR**	**95% CI**	**OR**	**95% CI**	**OR**	**95% CI**
**Sex** ^**1**^						
Boys	1		1		1	
Girls	2.28	1.82-2.84	2.20	1.60-3.04	4.18	2.59-6.76
**Age distribution** ^**2**^						
13–15 years	1		1		1	
≥16 years	1.02	0.83-1.26	1.36	1.01-1.84	0.73	0.50-1.08
**Family economy** ^3^					
Equal	1		1		1	
Worse	1.93	1.42-2.62	2.20	1.44-3.36	2.04	1.19-3.50
Better	0.88	0.65-1.20	0.84	0.53-1.35	0.51	0.24-1.06
**SPAI-C** ^3^	4.17	3.64-4.78	4.16	3.47-4.99	4.99	3.98-6.26
**SCL-5** ^3^	3.25	2.79-3.79	3.36	2.72-4.15	3.49	2.70-4.50
**Difficulties falling asleep** ^3^						
Occasionally/never	1		1		1	
Almost every night/often	2.33	1.85-2.95	2.49	1.78-3.48	2.40	1.57-3.68
**Early morning awakening** ^3^						
Occasionally/never	1		1		1	
Almost every night/often	2.67	1.96-3.64	3.38	2.24-5.12	2.03	1.09-3.76
**Self-rated health** ^3^						
Very good/good	1		1		1	
Not very good/poor	2.52	1.94-3.26	2.46	1.69-3.57	2.44	1.51-3.97
**Help seeking** ^3^				
No help seeking	1		1		1	
Psychologist	3.80	2.76-5.24	4.67	3.05-7.15	4.65	2.72-7.98
School health service	1.15	0.89-1.47	0.97	0.66-1.43	1.65	1.07-2.53
Doctor at hospital	1.35	1.08-1.69	1.23	0.88-1.72	1.72	1.14-2.59
All	1.44	1.09-1.90	1.48	0.97-2.26	3.41	1.75-6.64
**Physical activity** ^3^						
High	1		1		1	
Moderate	1.87	1.43-2.45	1.65	1.10-2.49	3.06	1.78-5.26
Low	2.63	1.99-3.46	2.82	1.90-4.21	3.50	1.98-6.20
**Alcohol intoxications** ^3^						
Never	1		1		1	
1-10 times	0.72	0.54-0.94	0.53	0.34-0.81	0.79	0.49-1.28
>10 times	0.51	0.37-0.71	0.63	0.40-0.98	0.28	0.13-0.60
**Smoking** ^3^					
Never/previous smoker	1		1		1	
Current smoker	1.14	0.85-1.52	1.16	0.77-1.74	1.23	0.71-2.13

1) Adjusted only for age.

2) Adjusted only for sex.

3) Adjusted for age and sex.

In addition, there was also an error in **Materials and methods**, *Measures*, “Social Anxiety Disorder Screening and Clinical Interview (n = 6,610 and 212)”, “Anxiety Disorders Interview Schedule for DSM IV: Child Version”, Paragraph 1. The number of participants who were asked the three social anxiety items was incorrectly stated as “n = 6,6610”, but should be “n = 6,610”. The corrected paragraph appears below.

The Anxiety Disorders Interview Schedule for DSM IV: Child Version is a semi-structured interview used to diagnose anxiety disorders and other mental disorders in children and adolescents, according to the DSM-IV criteria (American Psychiatric Association, 2000; Rasmussen and Neumer, 2015). In the present study, the interview modules for SAD, generalized anxiety disorder (GAD), separation anxiety disorder (SEP), specific phobias (SPH), obsessive-compulsive disorder (OCD), post-traumatic stress disorder (PTSD), dysthymia, and depression were used. For more convenient administration and coding, the modules were slightly shortened. In addition, questions regarding symptoms of substance abuse were asked, yet a diagnostic evaluation of substance abuse cannot be set based on the ADIS-C interview alone (Rasmussen and Neumer, 2015). The original version of ADIS-C has shown promising reliability (Lyneham et al., 2007), whereas research on psychometric properties of the Norwegian version is limited (Rasmussen and Neumer, 2015). However, the instrument is widely used in specialist health service, and items largely resemble the diagnostic criteria described in DSM-IV. It is highly recommended that it is used/applied only by trained clinicians with knowledge to the instrument and the diagnostic criteria (Rasmussen and Neumer, 2015). All participants (*n* = 6,610) were asked the following three social anxiety items from the ADIS-C (yes/no): “When you are with others, at school, in restaurants or at parties, do you ever feel that people might think that something you do is stupid or dumb?”, “When you are with other people at school, restaurants, or parties, do you think that people might laugh at you?”, and “When you are in these situations with others (school, restaurants, and parties), do you worry that you might do something that will make you feel ashamed or embarrassed?”. Individuals who answered yes to one or more questions were considered SP (*n* = 388) and invited to participate in a complete ADIS-C interview performed by specially trained psychiatric nurses. Those who answered no to all three questions were considered screening negative (SN; *n* = 6,222).

The authors apologize for these errors and state that this does not change the scientific conclusions of the article in any way. The original article has been updated.

